# Ventricular longitudinal shortening is an independent predictor of death in heart failure patients with reduced ejection fraction

**DOI:** 10.1038/s41598-021-99613-1

**Published:** 2021-10-13

**Authors:** J. Berg, R. Jablonowski, M. Mohammad, K. Solem, R. Borgquist, E. Ostenfeld, H. Arheden, M. Carlsson

**Affiliations:** 1 Clinical Physiology, Department of Clinical Sciences Lund, Lund University, Skåne University Hospital, Lund, Sweden; 2 Syntach AB, Lund, Sweden; 3 Cardiology, Department of Clinical Sciences Lund, Lund University, Skåne University Hospital, Lund, Sweden

**Keywords:** Epidemiology, Outcomes research, Prognostic markers, Heart failure

## Abstract

Reduced ventricular longitudinal shortening measured by atrioventricular plane displacement (AVPD) and global longitudinal strain (GLS) are prognostic markers in heart disease. This study aims to determine if AVPD and GLS with cardiovascular magnetic resonance (CMR) are independent predictors of cardiovascular (CV) and all-cause death also in heart failure with reduced ejection fraction (HFrEF). Patients (n = 287) were examined with CMR and AVPD, GLS, ventricular volumes, myocardial fibrosis/scar were measured. Follow-up was 5 years with cause of death retrieved from a national registry. Forty CV and 60 all-cause deaths occurred and CV non-survivors had a lower AVPD (6.4 ± 2.0 vs 8.0 ± 2.4 mm, p < 0.001) and worse GLS (− 6.1 ± 2.2 vs − 7.7 ± 3.1%, p = 0.001). Kaplan–Meier analyses displayed increased survival for patients in the highest AVPD- and GLS-tertiles vs. the lowest tertiles (AVPD: p = 0.001, GLS: p = 0.013). AVPD and GLS showed in univariate analysis a hazard ratio (HR) of 1.30 (per-mm-decrease) and 1.19 (per-%-decrease) for CV death. Mean AVPD and GLS were independent predictors of all-cause death (HR = 1.24 per-mm-decrease and 1.15 per-%-decrease), but only AVPD showed incremental value over age, sex, body-mass-index, EF, etiology and fibrosis/scar for CV death (HR = 1.33 per-mm-decrease, p < 0.001). Ventricular longitudinal shortening remains independently prognostic for death in HFrEF even after adjusting for well-known clinical risk factors.

## Introduction

Heart failure (HF) is a debilitating condition that affected an estimated 6.5 million Americans over the age of 20 in 2012, and the prevalence is projected to increase 46% by 2030, making HF a major challenge for the health-care system^1^. Reduced ventricular longitudinal shortening has been associated with progressive HF^2–4^, and it has been shown to be an earlier and more sensitive marker for contractile dysfunction than the commonly-used ejection fraction (EF)^5,6^. Patients with preserved EF can exhibit reduced longitudinal shortening^7,8^, illustrating a disadvantage of using EF as the sole marker of ventricular function. Additionally, experimental data show that subendocardial damage elicits isolated reductions in ventricular longitudinal shortening irrespective of EF^9^. Therefore, there could be advantages in considering longitudinal function when assessing cardiac function, rather than relying solely on common measurements such as EF.

Ventricular longitudinal shortening can be measured by atrioventricular plane displacement (AVPD)^10^ and global longitudinal strain (GLS)^11^. AVPD measures the average distance the atrioventricular (AV) plane moves towards the apex and is the major contributor to ventricular emptying and concomitantly to atrial filling during systole^12^. GLS is another well-known marker of ventricular longitudinal shortening^13^, and is a measure of the average segmental myocardial deformation. While AVPD and GLS are both measures of longitudinal shortening, the two cannot be used interchangeably^14–16^.

Both AVPD and GLS are reduced in patients with HF^17–19^. They are predictors of major adverse cardiac events and all-cause mortality in outpatients referred for clinical cardiac magnetic resonance (CMR)^20^, for patients with EF below 50%^21,22^ and for patients with hypertension^16^. However, it is not known whether selective assessment of ventricular longitudinal shortening in heart failure patients with reduced ejection fraction (HFrEF) adds prognostic value, since these patients are already known to have poor prognosis. Ventricular longitudinal shortening measurements are not presently included in standard clinical assessment of heart failure patients^23,24^ undergoing echocardiography or CMR. Clarifying the potential added benefit of ventricular longitudinal shortening over commonly-used parameters could aid in the clinical assessment of heart failure patients.

Previous studies have reported differences in the prognostic value of ventricular longitudinal shortening that can be explained by differences between populations and follow-up periods^16,20–22^. Moreover, different methods for assessment of ventricular longitudinal shortening have been used. The earliest studies on the prognostic value of AVPD using echocardiography measured AVPD in several positions^25–27^. Later studies using CMR focused on a single point in the lateral^16,20,21^ or septal wall^28^, or normalized AVPD to cardiac length^3^. While regional longitudinal strain is already known to be related to outcome^29,30^, it is unknown whether the prognostic value of AVPD is influenced by the location of the AVPD measurements in heart failure patients. This may in part explain why there is no recommended or standardized position for measuring AVPD using CMR imaging. However, six points encompassing the left ventricular AV-plane are available for analysis when using the three standard long-axis views in CMR.

Therefore, the primary aim of this study was to assess the prognostic value of ventricular longitudinal shortening variables as independent predictors of cardiovascular (CV) and all-cause death in a cohort of patients with HFrEF. Secondly, this study aimed to evaluate ventricular longitudinal shortening as a prognostic tool independent of well-known risk markers such as EF and late gadolinium enhancement (LGE). Thirdly, we aimed to evaluate the relative prognostic differences between measuring one point or using several averaged points encircling the AV-plane, for CV and all-cause death in this population.

## Results

### Baseline patient characteristics

Out of 295 patients with HFrEF undergoing a clinical CMR examination eight patients with poor image quality were excluded and so 287 patients were included for the final analysis (62 ± 13 years, 78% men). Figure S1 shows a flowchart of the study cohort. Patient characteristics are displayed in Table 1 for the whole group as well as stratified by AVPD-tertiles and GLS-tertiles. A total of 41% had ischemic cardiomyopathy (ICM) and 59% non-ischemic cardiomyopathies (NICM). Most patients had normal blood pressure (70%), were non-smokers (82%) and about a fifth had diabetes (22%). The patients were on optimal medical HF treatment with beta blockers (92%) and renin–angiotensin–aldosterone system (RAAS) inhibitors (90%), and a majority had diuretics, aspirin and spironolactone.

**Table 1:** Baseline characteristics stratified by AVPD and GLS tertiles.

Characteristics	Total	AVPD tertiles			GLS tertiles		
	n = 287	> 8.8 mm	6.8–8.8 mm	< 6.8 mm	< − 8.4%	− (8.4–6.1) %	> − 6.1%
Age (years)	62 ± 12	63 ± 12	63 ± 12	59 ± 14	62 ± 12	63 ± 12	60 ± 14
BMI (kg/m^2^)	27 ± 4	28 ± 5	27 ± 4	26 ± 5	27 ± 5	27 ± 4	27 ± 4
Male	225 (78)	67 (71)	73 (75)	85 (89)	63 (66)	76 (81)	82 (87)
ICM	168 (59)	51 (54)	63 (65)	54 (57)	51 (54)	61 (65)	53 (56)
Smoking	45 (18)	18 (22)	8 (9)	19 (22)	18 (21)	10 (12)	17 (20)
Hypertension	77 (30)	29 (34)	31 (35)	17 (20)	29 (33)	23 (27)	22 (26)
Diabetes	57 (22)	14 (17)	22 (24)	21 (24)	13 (15)	24 (28)	17 (20)
**NYHA**							
I	20 (9)	14 (20)	4 (6)	2 (3)	13 (17)	4 (6)	3 (4)
II	63 (29)	31 (44)	17 (24)	15 (20)	24 (32)	28 (41)	9 (13)
III	101 (47)	25 (35)	36 (51)	40 (53)	31 (41)	29 (42)	40 (59)
IV	32 (15)	1 (1)	13 (19)	18 (24)	8 (11)	8 (12)	16 (24)
Betablockers	241 (92)	75 (88)	83 (92)	83 (97)	80 (92)	78 (92)	80 (94)
RAAS antagonists	236 (90)	77 (91)	83 (92)	76 (88)	78 (90)	79 (93)	75 (88)
Spironolactone	133 (52)	36 (43)	48 (53)	49 (58)	41 (48)	39 (46)	51 (61)
eGFR (ml/min/1.73 m^2^)	68 ± 20	71 ± 18	66 ± 21	69 ± 21	68 ± 20	69 ± 19	68 ± 22
NT-proBNP (ng/l), median (IQR)	1604 (695–3405)	596 (333–1501)	1730 (740–3008)	2531 (1416–5469)	737 (430–1697)	1728 (583–3417)	2531 (1416–5508)
LGE presence	220 (77)	69 (73)	75 (77)	76 (80)	67 (71)	77 (82)	72 (77)
EDV (ml)	300 ± 93	278 ± 75	292 ± 90	327 ± 106	261 ± 77	291 ± 74	348 ± 106
ESV (ml)	225 ± 88	193 ± 62	214 ± 82	269 ± 99	178 ± 62	216 ± 63	283 ± 101
SV (ml)	75 ± 22	87 ± 19	77 ± 19	61 ± 18	83 ± 21	76 ± 21	66 ± 20
CO (l/min)	4.7 ± 1.5	5.4 ± 1.4	4.7 ± 1.3	3.9 ± 1.6	5.1 ± 1.5	4.7 ± 1.2	4.1 ± 1.7
EDV index (ml/m^2^)	150 ± 43	138 ± 32	148 ± 43	165 ± 50	131 ± 33	147 ± 36	174 ± 49
ESV index (ml/m^2^)	113 ± 42	95 ± 27	109 ± 39	135 ± 47	89 ± 27	109 ± 31	142 ± 48
SV index (ml/m^2^)	38 ± 10	43 ± 9	39 ± 9	31 ± 9	42 ± 9	38 ± 10	33 ± 10
CI (l/min/m^2^)	2.4 ± 0.7	2.7 ± 0.7	2.4 ± 0.6	2.0 ± 0.8	2.6 ± 0.7	2.4 ± 0.6	2.1 ± 0.8
Left atrial volume (ml)	127 ± 45	122 ± 43	119 ± 42	140 ± 48	118 ± 48	122 ± 38	142 ± 46
EF (%)	27 ± 8	32 ± 6	28 ± 7	20 ± 6	33 ± 6	27 ± 6	20 ± 7
Mean AVPD (mm)	7.8 ± 2.4	10.4 ± 1.4	7.7 ± 0.6	5.1 ± 1.1	9.8 ± 1.9	7.9 ± 1.6	5.5 ± 1.6
GLS (%)	− 7.5 ± 3.0	− 10.0 ± 1.4	− 7.5 ± 1.9	− 5.1 ± 2.1	− 10.8 ± 2.1	− 7.2 ± 0.7	− 4.4 ± 1.4
TAPSE (mm)	18.5 ± 6.3	22.7 ± 5.0	18.3 ± 5.6	14.5 ± 5.5	20.5 ± 6.1	19.2 ± 6.1	15.5 ± 5.6

Data are expressed as mean ± standard deviation or as absolute numbers and percentages in parentheses unless otherwise stated.

*BMI* body mass index, *ICM* ischemic cardiomyopathy, *NYHA* New York Heart Association, *RAAS* renin–angiotensin–aldosterone-system, *IQR* inter-quartile range, *LGE* late gadolinium enhancement, *EDV* end-diastolic volume, *ESV* end-systolic volume, *SV* stroke volume, *CO* cardiac output, *CI* cardiac index, *EF* ejection fraction, *AVPD* atrioventricular plane displacement, *GLS* global longitudinal strain, *TAPSE* tricuspid annular plane systolic excursion.

Four patients had insufficient image quality for strain analysis and were excluded, hence GLS could be measured in 283 out of 287 (99%) patients. Ventricular longitudinal shortening was lower in patients (AVPD 7.8 ± 2.4 mm and GLS − 7.5 ± 3.0%) compared with age-matched controls (AVPD 15.3 ± 1.6 mm and GLS − 20.6 ± 2.0%; p < 0.001 for both). Patients had larger volumes (EDV 300 ± 93 ml, ESV 225 ± 88 ml), smaller stroke volumes (75 ± 22 ml) and EF (26.5 ± 8.0%) than controls (EDV 163 ± 37 ml, ESV 66 ± 21 ml, SV 97 ± 20 ml, EF 59.8 ± 5.2%, p < 0.001 for all). LGE was present in 77% of patients, and out of the LGE positive patients, ICM (74%) was more commonly seen than NICM (26%).

The intraobserver variability was 0.5 ± 0.8 mm for AVPD [Intra class correlation (ICC): 0.95 (0.91–0.97)] and 0.2 ± 1.4% for GLS [ICC: 0.90 (0.82–0.95)]. The interobserver variability was 0.2 ± 1.0 mm for AVPD [ICC: 0.92 (0.82–0.95)] and 0.1 ± 1.4% for GLS [ICC: 0.90 (0.82–0.94)]. Bland and Altman-plots are presented in the Appendix (Figure S2).

### AVPD, GLS and NYHA classification

**Figure 1:**
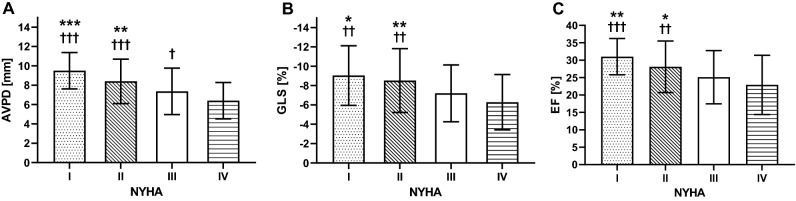
Ventricular function vs NYHA classification. (**A**) Atrioventricular plane displacement (AVPD); (**B**) global longitudinal strain (GLS); and (**C**) ejection fraction (EF) values stratified by New York Heart Association (NYHA) classes. There are stepwise reductions in AVPD, GLS and EF with increasing NYHA class and AVPD was the only variable that significantly differed between NYHA III and IV. Bars and whiskers represent means and standard deviations. *p < 0.05 vs NYHA III, **p < 0.01 vs NYHA III, ***p < 0.001 vs NYHA III, ^†^p < 0.05 vs NYHA IV, ^††^p < 0.01 vs NYHA IV, ^†††^vs NYHA IV.

Almost half (47%) of patients with available New York Heart Association (NYHA) classifications (n = 216) were in NYHA III (Table 1). There were stepwise reductions in AVPD, GLS and EF with increasing NYHA class and AVPD was the only variable that differed between NYHA III and IV (Fig. 1). Supplemental Table S1 presents values of AVPD, GLS and EF for each NYHA class.

### Survival analyses

The full follow-up time was median 7.9 years (IQR 5.4–11.7 years) and the first 5-year follow up showed 40 CV deaths and 60 all-cause deaths (data from the full follow-up are presented in Appendix). AVPD was higher in CV-death survivors than in non-survivors (8.0 ± 2.4 vs 6.4 ± 2.0 mm, p < 0.001). Likewise, GLS and EF was also higher in CV-death survivors than in non-survivors (− 7.7 ± 3.1 vs − 6.1 ± 2.2%, p < 0.001 and 27.1 ± 8.1 vs 22.6 ± 8.0%, both p = 0.001). Mean tricuspid annular plane systolic excursion (TAPSE) was 18.5 ± 6.3 mm and survivors had 3.4 mm greater amplitude than non-survivors (p < 0.001). Mean left atrial volume was 127 ± 45 ml and did not significantly differ between survivors and non-survivors (Δ10 ml, p = 0.22). Similar results were seen for all-cause death.

**Figure 2:**
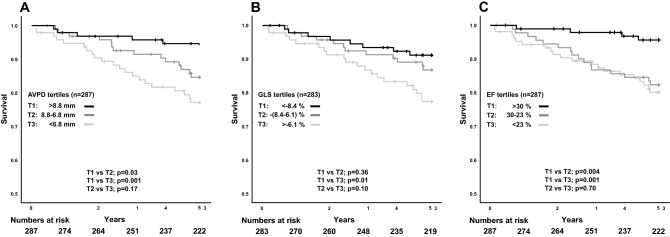
Cardiovascular death. Kaplan–Meier curves showing survival from cardiovascular death stratified by tertiles for (**A**) atrioventricular plane displacement (AVPD), (**B**) global longitudinal strain (GLS) and (**C**) ejection fraction (EF) for the 5-year follow-up in panels. *T1* upper tertile, *T2* middle tertile, *T3* lower tertile.

**Figure 3:**
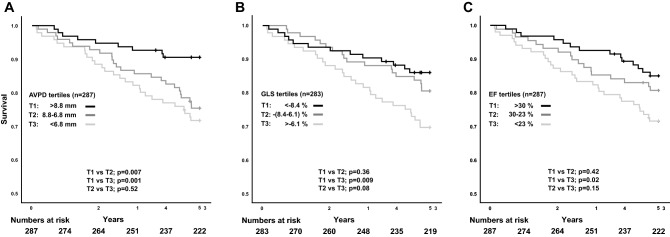
All-cause death. Kaplan–Meier curves showing survival from all-cause death stratified by tertiles for (**A**) atrioventricular plane displacement (AVPD), (**B**) global longitudinal strain (GLS) and (**C**) ejection fraction (EF) for the 5-year follow-up. *T1* upper tertile, *T2* middle tertile, *T3* lower tertile.

Additionally, CV deaths occurred to a higher extent in lower tertiles. The Kaplan–Meier plots show that patients in the highest AVPD tertile had better CV survival than the two lower AVPD tertiles both for CV death (Fig. 2A) and all-cause death (Fig. 3A). For GLS, only the highest tertile had better CV and all-cause survival compared with the lowest tertile (Figs. 2B, 3B), but no difference was shown between the two mid and low tertiles or between high and mid tertiles. EF tertiles showed similar results as AVPD but lacked separation between the mid and low tertile for CV death (Fig. 2C). Like GLS, only the highest EF tertile had better all-cause survival compared with the lowest tertile (Fig. 3C). Figure S3 and S4 show Kaplan–Meier plots of full follow-up for CV and all-cause death, respectively.

### AVPD and GLS as univariate predictors

In the univariate Cox regression analyses (Table 2), AVPD and GLS were both predictors of CV death (AVPD: HR 1.30 and GLS: HR 1.19) and all-cause death (AVPD: HR 1.21 and GLS: 1.14). When adjusting mean AVPD to LV length, HR was still significant (CV death: HR 1.27, p = 0.001 and all-cause death: HR 1.19, p = 0.002). Dichotomous presence of known clinical risk factors such as etiology of decreased EF, presence of LGE, and diabetes, showed, as expected, a high HR for CV death. Of note, at the 5-year follow-up patients with ICM had a 2.5 times higher risk of CV death than those with NICM. Patients with diabetes were similarly at high risk of CV death (HR 2.39). Presence of LGE on CMR was associated with an almost three-fold increased risk of CV death. In contrast, presence of, or treatment for, hypertension resulted in a 4 times lower risk of CV death (HR 0.25) and a lower, albeit not significant, risk of all-cause death (HR 0.66, p = 0.24) at the 5-year follow-up. TAPSE (per-mm-decrease) was a univariate predictor of CV death (HR 1.09, p < 0.001) and left atrial volume was not (HR 1.04, p = 0.23). Table S2 shows univariate predictors at full follow-up.

**Table 2:** Univariate predictors for the 5-year follow-up.

Univariate predictors for HFrEF				
Variable	Cardiovascular death		All-cause death	
	HR (95% CI)	p value	HR (95% CI)	p value
Age, per 1 year	**1.05 (1.02–1.09)**	**0.001**	**1.04 (1.02–1.08)**	**0.001**
Sex, Male	1.64 (0.69–3.91)	0.26	1.88 (0.89–3.96)	0.96
BMI, per 1 kg/m^2^	0.93 (0.86–1.01)	0.08	**0.93 (0.87–0.99)**	**0.03**
Diabetes	**2.39 (1.13–5.02)**	**0.02**	**3.05 (1.71–5.45)**	**< 0.001**
NYHA	1.54 (0.99–2.39)	0.06	**1.57 (1.07–2.31)**	**0.02**
NT-proBNP, per 1000 ng/l	**1.17 (1.05–1.30)**	**0.004**	**1.17 (1.10–1.25)**	**< 0.001**
eGFR, per 10 ml/min/1.73 m^2^	**0.81 (0.68–0.97)**	**0.02**	**0.79 (0.69–0.92)**	**0.002**
Smoking	0.91 (0.35–2.39)	0.85	0.86 (0.39–1.94)	0.72
Hypertension	**0.25 (0.08–0.82)**	**0.02**	0.66 (0.33–1.33)	0.24
Etiology, ICM	**2.59 (1.23–5.45)**	**0.01**	**2.26 (1.26–4.05)**	**0.006**
LGE presence	**2.89 (1.03–8.12)**	**0.04**	**2.43 (1.11–5.35)**	**0.03**
Left atrial volume, per 10 ml	1.04 (0.98–1.11)	0.23	1.02 (0.97–1.08)	0.51
EF, per 1%	**1.06 (1.02–1.11)**	**0.002**	**1.04 (1.003–1.07)**	**0.03**
EDVi, per 10 ml/m^2^	**1.06 (1.01–1.13)**	**0.04**	1.03 (0.97–1.09)	0.27
Mean AVPD, per 1 mm	**1.30 (1.13–1.49)**	**< 0.001**	**1.21 (1.09–1.36)**	**0.001**
GLS, per 1%	**1.19 (1.07–1.33)**	**0.002**	**1.14 (1.04–1.25)**	**0.004**
TAPSE, per 1 mm	**1.09 (1.03–1.14)**	**0.001**	**1.09 (1.04–1.13)**	**< 0.001**

Bold text indicates statistical significance.

*HR* hazard ratio, *CI* confidence interval, *BMI* body mass index, *NYHA* New York Heart Association, *BNP* brain natriuretic peptide, *eGFR* estimated glomerular filtration rate, *ICM* ischemic cardiomyopathy, *LGE* late gadolinium enhancement, *EF* ejection fraction, *EDVi* end-diastolic volume indexed to body surface area, *AVPD* atrioventricular plane displacement, *GLS* global longitudinal strain, *TAPSE* tricuspid annular plane systolic excursion.

### AVPD and GLS as independent predictors

AVPD persisted as an independent predictor of CV death (HR of 1.33-per-mm-decrease) at the 5-year follow-up when adjusting for well-known clinical risk factors (age, sex, body mass index (BMI), etiology of decreased EF, LGE presence, EF and EDVi) in multivariate Cox regression analysis (Table 3). There was a significantly better model prediction of CV death when adding AVPD to the aforementioned risk factors (log-likelihood ratio (LR) test; χ^2^(1) = 17.0, p < 0.001) (Table 3). Also, AVPD was an independent predictor of all-cause death (HR 1.24) and added incremental prognostic value (LR test; χ^2^(1) = 14.6, p < 0.001) over the clinical risk factors.

**Table 3:** Multivariate modeling for death in HFrEF.

Multivariate Cox regression 5-year follow-up	Cardiovascular death		All-cause death	
	HR (95% CI)	p value	HR (95% CI)	p value
**Model 1—AVPD (n = 287)**	**LR test p < 0.001**		**LR test p < 0.001**	
Age, per 1 year	**1.06 (1.02–1.09)**	**0.001**	**1.04 (1.02–1.07)**	**< 0.001**
Sex, male	1.11 (0.44–2.77)	0.83^1^	1.36 (0.63–2.91)	0.44^4^
BMI, per 1 kg/m^2^	0.96 (0.88–1.04)	0.29^4^	0.94 (0.88–1.01)	0.06^6^
Etiology, ICM	1.93 (0.86–4.37)	0.11^5^	0.63 (0.34–1.17)	0.15^5^
LGE presence	1.40 (0.43–4.56)	0.58^2^	1.34 (0.53–3.35)	0.54^3^
EF, per 1%	1.02 (0.96–1.09)	0.47^3^	1.00 (0.95–1.05)	1.00^1^
EDVi, per 10 ml/m^2^	1.05 (0.98–1.13)	0.15^6^	1.01 (0.94–1.08)	0.78^2^
Mean AVPD, per 1 mm	**1.33 (1.16–1.53)**	**< 0.001**	**1.24 (1.11–1.39)**	**< 0.001**
**Model 2—GLS (n = 283)**	LR test p = 0.26 (GLS not significant)		**LR test p = 0.002**	
Age, per 1 year	**1.06 (1.03–1.10)**	**< 0.001**	**1.04 (1.02–1.07)**	**0.001**
Sex, male	0.95 (0.38–2.37)	0.91^1^	1.31 (0.61–2.84)	0.49^3^
BMI, per 1 kg/m^2^	0.95 (0.88–1.03)	0.24^5^	0.94 (0.88–1.01)	0.08^6^
Etiology, ICM	1.87 (0.85–4.01)	0.12^6^	1.68 (0.89–3.17)	0.11^5^
LGE presence	1.49 (0.45–4.90)	0.52^3^	1.26 (0.49–3.24)	0.63^2^
EF, per 1%	**1.08 (1.04–1.13)**	**< 0.001**	1.02 (0.98–1.06)	0.28^4^
EDVi, per 10 ml/m^2^	1.01 (0.93–1.10)	0.82^2^	0.99 (0.91–1.06)	0.72^1^
GLS, per 1%	1.07 (0.93–1.23)	0.37^4^	**1.15 (1.05–1.25)**	**0.003**

Bold text indicates statistical significance.

*HR* hazard ratio, *CI* confidence interval, *LR* likelihood ratio, *BMI* body mass index, *NYHA* New York Heart Association, *ICM* ischemic cardiomyopathy, *LGE* late gadolinium enhancement, *EF* ejection fraction, *EDVi* end-diastolic volume indexed to body surface area, *AVPD* atrioventricular plane displacement, *GLS* global longitudinal strain. Log-likelihood ratio (LR)-tests assess the added prognostic values compared with nested models without AVPD (Model 1) and GLS (Model 2). Numbers in superscript represents the last step before elimination for that variable.

GLS did not remain as an independent predictor of CV death at the 5-year follow-up as it was dropped in the model selection (Table 3). However, GLS was an independent predictor of all-cause death after adjusting for the same risk factors. Inclusion of GLS and AVPD in the same stepwise multiple regression canceled the predictive value of GLS, as GLS and AVPD were co-linear with a regression coefficient of R = 0.76 (p < 0.001). Of note, the linear correlation between AVPD and EF was significant (R = 0.66, p < 0.001), as was the correlation between GLS and EF (R = 0.65, p < 0.001). Table S3 shows multivariate modeling with AVPD and GLS at full follow-up. Additionally, TAPSE was an independent predictor adjusted for the a priori clinical risk factors when used in place of AVPD or GLS (HR 1.06, p = 0.046, Table S4).

### Prognostic values of different AVPD measurement points

**Figure 4:**
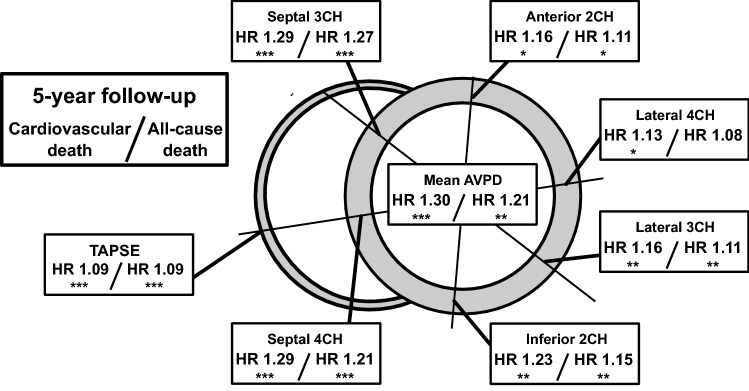
Prognostic values by measurement location. Univariate Cox regression analyses of the six different atrioventricular plane displacement (AVPD) locations. The short-axis slice illustrates hazard ratios (HR) for cardiovascular death and all-cause death at the 5-year follow-up for each location. Septal and average locations indicate higher HR values than lateral, anterior and inferior points. Asterisks represent *p < 0.05, **p < 0.01, ***p < 0.001. *TAPSE* tricuspid annular plane systolic excursion.

Univariate Cox regression analyses for CV death for all six AV-plane locations for the 5-year follow-up are presented in Fig. 4. The highest HR of a single AVPD location was obtained from the septal locations in both the 3- and 4-chamber views (CV death: HR 1.29 and 1.29 and all-cause death: HR 1.27 and 1.21, respectively). Figure S5 shows the univariate analyses at all AVPD locations at full follow-up.

## Discussion

The main findings of this study are threefold. Firstly, the ventricular longitudinal shortening parameters AVPD and GLS were univariate predictors of CV and all-cause death in HF patients with severely reduced systolic function (EF < 40%) at 5 years. Secondly, AVPD was, unlike GLS, an independent prognostic marker for CV death at 5 years even after adjusting for well-known clinical risk factors such as EF, age, sex, BMI, etiology of decreased EF, presence of LGE and EDVi. Both AVPD and GLS, however, persisted as independent predictors of all-cause death. Thirdly, septal AVPD and mean AVPD resulted in the highest values of HR for CV and all-cause death, indicating that the prognostic value of AVPD is influenced by measurement location. It is not evident that GLS or AVPD would have prognostic importance when the global function measured as EF is severely reduced and the novelty of the present study is showing that ventricular longitudinal shortening retains its independent prognostic value in this patient population.

### Prognostic relevance of AVPD and GLS in HFrEF

This study specifically targeted heart failure with severely reduced EF, in which AVPD and GLS are known to already be reduced^13,18,25^. The prognostic link between ventricular longitudinal shortening and death was first shown in echocardiography in heart failure patients^17^ and in patients hospitalized with acute myocardial infarctions^31^. Our study adds information about the prognostic relationship between AVPD, GLS and death in patients with severe HF, as these latter studies focused on cohorts with less disease severity. Previous CMR studies have investigated mitral annular plane systolic excursion (MAPSE) as a predictor of major adverse cardiovascular events in unselected outpatients referred for clinical CMR (HR 1.34 per-2-mm-decrease)^20^ and in patients with ST-elevation myocardial infarction (HR 1.20 per-mm-decrease)^28^. Furthermore, CMR-derived MAPSE has been shown as a predictor of all-cause death in patients with EF < 50% (HR 1.65 per-mm-decrease)^21^ and in patients with hypertension (HR 1.47 per-mm-decrease)^16^. While these studies had follow-up times with a span from 1.5 to 5 years, we found HR for AVPD at 5 years in the same range (HR 1.33) in our cohort of patients with severe disease.

There can be several factors responsible for the variations in prognostic values obtained in the previous studies. Firstly, these studies encompassed different populations with different levels of disease severity and mortality. Secondly, the different follow-up time can be a factor. And thirdly, the location of measurement may play a role.

#### Population factors

At our 5-year follow-up, we had a total mortality of 21% which is about 40% higher than the three studies from Romano et al.^16,21,22^. In these three populations, mean EF ranged from 33 to 59% compared to our population (EF 26.5 ± 8.0%) which could explain the difference in mortality. As the total number of CV and all-cause deaths (n = 40 and n = 60) relative to our population size (n = 287) are higher than previous CMR studies of ventricular longitudinal shortening, our power to perform multivariate modeling is adequate, even though the number of subjects is moderate. Our prognostic values (hazard ratios) are lower than previous CMR studies^16,20–22,28^, possibly because of the narrow range of AVPD and GLS in our high morbidity and mortality population.

#### Length of follow-up

The length of follow-up can also affect the prognostic values from ventricular longitudinal shortening variables. Longer follow-up carries more information and could aid in distinguishing prognostic information among groups with small differences. However, longer follow-up times expose the patients to an increasing number of confounding factors that are impossible to adequately control for. The Appendix includes results of the full follow-up time (median 7.9 years [5.4–11.7 years]) which unmask a non-proportional hazard of AVPD and GLS. The long-term data suggest a convergent behavior of AVPD and GLS tertiles after 10 years, as shown in the Kaplan–Meier curves. This means that the increased risk of CV and all-cause death in groups separated by ventricular longitudinal shortening evens out as time passes. The drop in HR from 5 years to full follow-up seen with AVPD and GLS confirms this convergence. There is a vast number of factors (e.g. increasing age, significant comorbidities, surgeries, lifestyle changes, accidents etc.) affecting the prognosis 10 + years after the initial CMR scan which can contribute to this finding. We have not found any other examples in the literature of prognostic studies regarding ventricular longitudinal shortening measurements with a similar length of follow-up able to verify this finding.

#### Measurement location

We found septal AVPD to have the highest HR of the single measurement locations. This is in line with Mayr et al.^28^, who found the septal point to be of higher predictive value than the lateral point. Rangarajan et al.^20^, however, did not find the septal position to be a significant predictor of major adverse cardiac events. The reasons for this were not clear to the authors and differs from our results. One methodological difference is that we measured a perpendicular distance from the AV-plane whereas Rangarajan et al. measured the direct route (beeline) of the mitral annular position from ED to ES. The effect of these methodological differences would, however, be expected to be small. In addition to the septal and lateral points, our study suggests that the six single AVPD locations influence the prognostic values in a slightly different way.

There was a slight insubstantial decrease, in HR by indexing AVPD to LV length in our study. The indexed AVPD is the equivalent of the “long-axis strain” presented by Gjesdal et al. in the Multi-Ethnic study of Atherosclerosis (MESA)^3^ and sometimes also referred to “long-axis shortening”. This is in concordance with the study by Støylen et al.^15^ showing that normalizing MAPSE to LV length did not adequately adjust for inter-individual size-dependent variability compared to only MAPSE, but rather that the method could add geometric biases.

In this study, worse GLS was associated with increased CV death rates, higher NYHA class, and was an independent marker for all-cause death. Both AVPD and GLS were independent predictors of CV death for the full follow-up (data in Appendix). There were small differences in the distribution of CV deaths during the 5-year follow-up when comparing AVPD and GLS. These differences were enough for AVPD to be a significant independent predictor of the main outcome. We do not believe that GLS as an important predictor of outcome in heart disease^22,32–34^ is contradicted by our results. Longitudinal strain tools have the possibilities of analyzing regional and segmental strain values, something that is advantageous compared to AVPD.

In contrast to our results Holzknecht et al*.*^35^ found GLS to be a superior prognostic predictor for a composite endpoint compared to MAPSE and EF in STEMI patients within one week of treatment. This may be explained by differences in patient populations and outcome measures between the studies. Additionally, our univariate results for GLS at 5 years was slightly lower but comparable to similar studies in patients with EF < 50% (HR 1.35)^22^ and dilated cardiomyopathy (HR 1.35)^34^.

Of note, the presence of hypertension was associated with a markedly decreased risk of CV death, as also shown by Zarrinkoub et al.^36^. Our data suggest this effect to be a short-term one since it disappears in the full follow-up data. Hypothetically, hypertension in HFrEF patients might indicate cardiac reserve capacities and a higher tolerance for medical treatments.

### Physiology of ventricular longitudinal shortening

Our study adds knowledge to the prognostic link between ventricular longitudinal shortening and death in a patient group with severe systolic dysfunction. In pathophysiological aspects, GLS measures the myocardial deformation throughout the myocardium while AVPD measures the result of that deformation on the AV-plane. The interaction between the AV-plane and atria is the direct measure of atrio-ventricular coupling and thus AVPD have a direct pathophysiological coupling to atrial filling in systole and to ventricular diastolic filling and may enhance the prognostic performance compared to GLS seen in this study.

Ventricular longitudinal shortening reflected in AVPD and GLS causes displacement of blood to the great arteries in systole. While both measurements generally are appraised by their systolic properties, their contribution to filling of the heart is also crucial for the maintenance of cardiac output and may in part explain the strong prognostic information in GLS and especially AVPD. The systolic movement of the mitral and tricuspid valves towards the apex ejects blood into the great arteries while simultaneously causing suction of blood into the atria^37^. This was corroborated by a recent CMR study measuring flow profiles in the pulmonary veins in both controls and heart failure patients^38^.

The pathophysiological connection between reduced ventricular longitudinal shortening and deaths related to heart failure is more easily understood than the connection between reduced ventricular longitudinal shortening and myocardial infarction (MI) or arrhythmias. One explanation may be that reduced AVPD and GLS show the effects from ischemic cardiomyopathy and prior ischemic insult. Thus, reduced ventricular longitudinal shortening may reflect damage to the myocardium not recognized by LGE. Of note, extracellular volume quantification was not available in our study population and may have provided prognostic information. Experimental data have shown that reduced AVPD after acute MI yields a concomitant decrease in SV^39^ which also helps to understand why ventricular longitudinal shortening would be prognostic in ischemic heart failure.

### Prognostic values of different AVPD measurement points—where to measure?

By including six AVPD measurement points we could evaluate relative differences in prognostic values. We found that the two septal and mean AVPD measurement points yielded the highest HR while the lateral positions the lowest. One explanation for this is that amplitudes of septal AVPD are inherently smaller than lateral AVPD^40^, and therefore a one-millimeter decrease is a relatively greater reduction at the septal compared to lateral position. This relative difference is important to consider when evaluating results from different outcome studies of ventricular longitudinal shortening for adequate comparisons. This might also be valuable information when choosing which measurements to use for a planned study. For the clinical setting, single-point septal AVPD seems to be a good choice, but a more diligent approach by averaging several measurements reduces the risk of outliers and may decrease variability. As automated post-processing algorithms for AVPD become available, the mean value from several long axis views may be the most robust approach, without being time-consuming, both for prognostic studies and in the clinical setting.

### Limitations

Since we had a long follow-up time and patients were included between 2003–2015, optimal medical treatment has changed over the inclusion period. Hence, patients diagnosed in the later part of the inclusion period could have received better medical treatment at baseline and this would improve their prognosis. However, there were no significant differences in medications for patients included when comparing three tertiles split by inclusion dates. The medical treatments prescribed at the time of CMR show that most patients had betablockers and RAAS antagonists but only about half received spironolactone. Forty-two % of patients had all three medications at the time of inclusion and we do not have data on changes in medical therapies. Nevertheless, the overall 5-year mortality rate in our study is in the same range (21%) as the reported average survival estimates of congestive HF in Sweden for similar age-groups for years 2006–2010 (22% for 50–59 years, 29% for 60–69 years)^36^. In sum, differences regarding medical therapies are likely to be of minor confounding importance.

Fifty-six % of the patients were planned for implantable cardioverter-defibrillator (ICD) or cardio-resynchronizing therapy (CRT) implantation. This can affect the prognoses as these devices have been shown to increase survival, especially within ischemic cardiomyopathies^41,42^. Additionally, the indications of cardio-resynchronizing therapy devices and cardioverter defibrillators has been expanded for HFrEF patients during the last two decades and the survival benefits of these devices are not included in our general models. Of note, as CRT and ICD treatment are commonly used in patients with HFrEF it is of importance to include these patients in prognostic studies.

A methodological factor to consider is measurement error. The inter and intra-observer variability were higher in GLS compared to AVPD measurements which could lower the predictive performance of GLS compared to AVPD and in part explain our results.

It can be challenging to determine if the cause of death is related to CV disease. HFrEF patients are prone to have or later on acquire other significant comorbidities such as chronic obstructive pulmonary disease, chronic kidney disease or cancer. These comorbidities impact survival, which is the case over a long follow-up time. The cause of fatal arrythmia could primarily originate from non-CV comorbidities rather than decreased longitudinal shortening. Furthermore, it is possible that there are more confounding factors from comorbidities which we haven’t addressed. Nevertheless, CV death as an endpoint yielded consistently higher hazard ratios than all-cause death to the heart failure-associated predictors studied here indicating CV death to be more sensitive to apply in prognostic studies of heart failure. Our data still suggest that measures of ventricular longitudinal shortening can significantly predict survival even when adjusting for well-known risk factors, and the Appendix includes a long follow-up time which allow analysis of long-term prognostics. Having small numbers of patients at the end of follow-up is often a limitation in survival analyses. We still had 95 patients included at year 10, but only 19 patients left at year 15 which warrants that cautious interpretation of the data between these last years.

## Conclusions

Measures of ventricular longitudinal shortening are important predictors of cardiovascular and all-cause death in patients with HFrEF, and remains independently prognostic even after adjusting for well-known clinical risk factors. AVPD measured in the septum and mean AVPD demonstrated the highest hazard ratio indicating that measurement location can influence the prognostic results. Measurements of AVPD are quick, do not require specialized software, and can be performed at one or several points, and therefore appear to be clinically more favorable than GLS for determining prognosis in patients with HFrEF.

## Methods

### Study population

Patients with HFrEF undergoing CMR at Lund University Hospital during 2003–2015 were included. All patients gave informed written consent to have their data used for research purposes prior to CMR examination. This study was approved in 2019 by the Swedish National Review Authority, the Swedish National Board of Health and Welfare and conformed to the principles of the Declaration of Helsinki. All methods were carried out in accordance with relevant guidelines and regulations. Previous medical history and cause of death was obtained from the Swedish National Board of Health and Welfare's Cause of Death Registry.

Patients were included if they had a clinical diagnosis of heart failure and a CMR examination. Indications for CMR examinations were assessments for infarct, fibrosis, ventricular volumes or investigation prior to ICD or CRT. Exclusion criteria were EF above 40% measured from the CMR examination or inadequate image quality. The etiology of decreased EF was divided into ICM or NICM based on patients’ charts and the clinical CMR reports. Information on patient medications, hypertension, diabetes and NYHA classifications were retrospectively obtained from electronic medical records. Twenty age-matched healthy controls recruited from advertisements were included for comparisons, and whose characteristics have previously been published in detail^43^. In short, healthy controls had no history of cardiovascular disease, diabetes or systemic disease, no cardiovascular medication, no hypertension (blood pressure < 140/90 mmHg), and no pathology on electrocardiography or CMR.

The primary outcome was cardiovascular death. CV death was defined as death caused by acute myocardial infarction, heart failure or ventricular arrhythmia as stated by the national registry. The secondary outcome was all-cause death.

### CMR protocol

Imaging was performed using three different clinical MRI-scanners:

1. 1.5T Philips Achieva (Best, the Netherlands)
2. 3T Philips Achieva (Best, the Netherlands)
3. 1.5T Siemens Aera (Erlangen, Germany)

Standard short and long axis cine images (2ch, 3ch and 4ch) were acquired using steady-state free precession sequences. LGE sequences for infarct assessment were used according to the clinical protocol. Detailed imaging parameters are presented in the Appendix.

### CMR image analysis for AVPD and GLS assessment

Left ventricular (LV) volumes and longitudinal measurements were analyzed from cine images using freely-available imaging analysis software (Segment 2.2, http://segment.heiberg.se)^44^.

**Figure 5:**
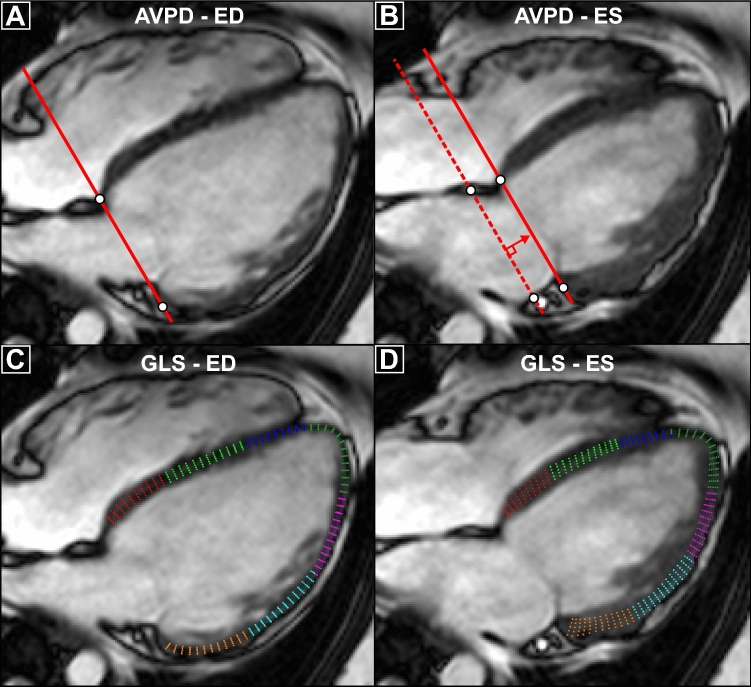
Measurement illustration. Illustration of how atrioventricular plane displacement (AVPD) and global longitudinal strain (GLS) were measured in a 4-chamber long-axis image. To obtain AVPD values (top row) annular annotation points are placed at the top of myocardium at end-diastole (ED, **A**) and at end-systole (ES, **B**). The perpendicular distance from the ED AV-plane to ES defines AVPD (red arrow, **B**). For GLS (bottom row) the myocardium is manually delineated in ED (**C**) and used as input in the semi-automated feature-tracking algorithm. The average longitudinal strain for each colored segment defines GLS.

AVPD was measured according to previously published methodology^12^ in each time frame using a validated semi-automatic tracking algorithm^45^. In short, annotation points were placed at the highest myocardial point in each long-axis view for the AV-plane and additional points for the apex in end-diastole (in total six measures of LV AV-plane). AVPD was defined as the mean displacement of these six LV points. Further, mean AVPD was indexed to LV length, and LV length was defined as the distance from the apex to the point intersecting the AV-plane at a perpendicular angle. The main advantages for using AVPD compared to the commonly used MAPSE are threefold. First, the basal measuring points for AVPD placed at the top of the ventricular muscle vs. at the mitral valve hinge-points for MAPSE provides easier tracking in CMR. Second, the six measuring points encircling the ventricle increases robustness to measurement errors compared to fewer measurement locations often used for MAPSE. Third, the perpendicular distance from an end-diastolic reference plane used for AVPD vs. the direct route often used in MAPSE circumvents possible biases via lateral translation of the ventricle and enables measuring volumes connected to longitudinal pumping. TAPSE was measured as the lateral free-wall displacement in the 4-chamber view. GLS was calculated using feature tracking in each long-axis view. The myocardium was manually delineated in end diastole and tracked semi-automatically and time-resolved over the cardiac cycle. If tracking was inadequate, it was manually adjusted in end diastole and re-tracked. Both AVPD and GLS measurements required at least two long-axis images for inclusion. Left atrial volume was estimated using the area-length method: V = (8/3π) × (Area2ch × Area4ch)/(L), where L is the shortest length from the 2ch or 4ch-view^46^. Figure 5 presents an example of AVPD and GLS measurements. Absence or presence of LGE and ischemic or non-ischemic etiology of LGE were visually determined by the attending physician.

### Follow-up

All included patients had follow-up data obtained from the Swedish National Health and Welfare’s Death Registry. Elapsed time between CMR examination and CV or all-cause death served as input for survival analyses. Patients were censored if they reached the end of the study period (31 December 2019) without an event. Patients who died from other causes were censored from the primary outcome at time of death. Follow-up analysis was performed for the 5-year follow-up, as the prognostic implication is expected to be most important in the first few years after the imaging test. Data from the complete follow-up time is presented in the Appendix.

### Statistical analysis

Continuous data are presented as mean ± standard deviation (SD) or median and interquartile range (IQR) according to normal distribution. Normal distribution was assessed by histograms. Categorical variables are presented as absolute numbers and proportion in percentages. Baseline characteristics were compared among equally-numbered AVPD- or GLS-tertiles with analysis of variance (ANOVA) and with least significant difference post-hoc tests. Differences between two groups were tested with Student’s *t* test for continuous variables and Chi-square test for categorical values. Intra, and interobserver variability for AVPD and GLS was tested in a subset of 43 patients with bias ± SD according to Bland and Altman^47^. Intra class correlation with a two-way-random-model was used to assess inter, and intra-observer reliability of the AVPD and GLS measurements.

Cumulative survival probability curves for CV death were assessed using Kaplan–Meier plots. The log-rank test was used for the 5-year follow-up. Cox regression modeling was used for calculation of hazard ratios (HR) of CV and all-cause death. HR are presented with 95% confidence intervals (95% CI). A priori defined well-known clinical risk factors for CV death [age, sex, BMI, etiology of decreased EF, presence of LGE, EF, end-diastolic volume indexed to body surface area (EDVi)] and with < 5% missing values were included in a multivariate Cox regression analysis. Multicollinearity between variables was assessed with Pearson’s linear regression coefficient with a cut-off value for inclusion chosen as < 0.8^48^. Schoenfeldt residuals plotted against time was used to assess proportional hazards. For the multivariate Cox regressions, a conditional backwards elimination strategy was performed, and a variable was included when p ≤ 0.10. Final predictive models with AVPD and GLS were compared with nested models including the aforementioned risk factors with the LR-test.

A p-value < 0.05 was considered significant. Statistical analyses were performed using SPSS v.27.0 (IBM Corporation, Armonk, NY, USA).

### Ethics approval and consent to participate

The patients gave informed written consent prior to the CMR examination that their data could be used for research purposes. Patients were able to retract their consent at any time. The patients’ data could also be used in retrospective cardiovascular research studies. This study was approved in 2019 by the Swedish Ethical Review Authority, the Swedish National Board of Health and Welfare in an amendment to the original form without new informed consent and conformed to the principles of the Declaration of Helsinki.

## Supplementary Information

Supplementary Information.

## Abbreviations

AVPD: Atrioventricular plane displacement
ANOVA: Analysis of variance
BMI: Body mass index
CMR: Cardiovascular magnetic resonance
CRT: Cardio-resynchronizing therapy
CV: Cardiovascular
EDVi: End-diastolic volume index
EF: Ejection fraction
GLS: Global longitudinal strain
HF: Heart failure
HFrEF: Heart failure with reduced ejection fraction
HR: Hazard ratio
ICD: Implantable cardioverter-defibrillator
ICM: Ischemic cardiomyopathy
IQR: Interquartile range
LGE: Late gadolinium enhancement
LR: Log-likelihood ratio
LV: Left ventricular
MAPSE: Mitral annular plane systolic excursion
NICM: Non-ischemic cardiomyopathy
NYHA: New York Heart Association
RAAS: Renin–angiotensin–aldosterone system
TAPSE: Tricuspid annular plane systolic excursion
