# Supplementary material for: Ventricular longitudinal shortening is an independent predictor of death in heart failure patients with reduced ejection fraction
Source: Sci Rep. 2021 Oct 13;11:20280. doi: 10.1038/s41598-021-99613-1 (PMC8514526; doi:10.1038/s41598-021-99613-1)
Supplement: Supplementary file 1 — Supplementary Information. [file 41598_2021_99613_MOESM1_ESM.docx]

**Appendix**

**Imaging parameters**

Imaging parameters for SSFP were typically; T_Echo_/T_Rep_ 1.5/3.0 ms and flip angle 60° (Achieva 1.5T), T_Echo_/T_Rep_ 1.8/3.7 ms and flip angle 45° (Achieva 3T) or T_Echo_/T_Rep_ 1.2/2.7 ms and flip angle 60° (Aera 1.5T). Ventricular volumes were measured from short axis delineations. Acquired resolution was typically 32 ms and reconstructed spatial resolution was typically 0.75x0.75x8 mm.

Late gadolinium enhancement (LGE) sequences for infarct assessment were used according to the clinical protocol. At Philips either a 2D PSIR or a 3D IR was used and on Siemens a 2D PSIR. Imaging parameter for LGE were typically; T_Echo_ 4 ms, effective repetition time every second heartbeat and flip angle 25° (Philips 2D PSIR), T_Echo_ 1.3 ms, effective repetition time every heartbeat and flip angle 15° (Philips 3D IR), T_Echo_ 3 ms, effective repetition time every second heartbeat and flip angle 25° (Siemens 2D PSIR). The inversion time was typically 280-350 ms.

**Statistical analysis** Cumulative survival probability curves for CV death were assessed using Kaplan-Meier plots and the generalized Wilcoxon test was used for the full follow-up to assess differences among survival curves. We chose to use the Generalized Wilcoxon test in place of the commonly used log-rank test since this is weighted so that time-points with more patients impacts the results more. Cox regression modelling was used for calculation of hazard ratios (HR) of CV and all-cause death. HR is presented with 95% confidence intervals (95%CI). A priori defined well-known clinical risk factors for CV death (age, sex, body mass index (BMI), aetiology of HF, presence of LGE, EF, end diastolic volume-indexed to body surface area (EDVi)) and with <5% missing values were included in a multivariate Cox-regression analysis. Including both EF and AVPD/GLS knowingly raises an issue of multicollinearity since all are surrogates of contractility. However, it is common for similar studies (e.g Rangarajan *et al.* JCMR 2016, Romano *et al*. Radiology 2017) to include EF into the multivariate analysis along with the variables of interest. To decrease the issue of overfitting by using a variable selection process to get more sparse models.

**Results**

***Survival analyses***

The 5-years follow-up in which 40 CV deaths and 60 all-cause deaths occurred resulted in median endpoint ratios of 2.1 and 2.0, respectively. The median full follow-up time was 7.9 years (interquartile range 5.4-11.7 years) and 83 CV deaths and 134 deaths occurred during this period. CV-death survivors had a median endpoint time of 9.0 years vs non-survivors of 5.2 years yielding a median endpoint ratio of 1.7. All-cause survivors had a median endpoint time of 9.9 years vs non-survivors of 5.4 years and a median endpoint ration of 1.8. ­­­­

AVPD and GLS were higher in CV-death survivors compared with non-survivors (8.0±2.5 vs 7.1±2.2 mm, p=0.005. -7.9±3.1 vs -6.6±2.6 %, p<0.001, respectively) but EF was not significantly higher in CV-death survivors (27.0±8.0 vs 25.1±7.9 %, p=0.08).

***AVPD and GLS as univariate predictors***

AVPD and GLS were both indicators of CV death (AVPD: HR 1.15 and GLS: HR 1.13) and all-cause death (AVPD: HR 1.12 and GLS: HR 1.10) at full follow-up, supplemental Table S1. AVPD adjusted to LV length did not cause a change in HR for CV death (HR 1.15, p=0.004) and a small decrease in all-cause death (HR 1.10, p=0.008).

***AVPD and GLS as independent predictors***

When adjusting for well-known clinical risk factors (age, sex, BMI, etiology of HF, LGE presence, EF and EDVi) in multivariate cox-regression modelling, AVPD as a continuous variable remained an independent predictor of both CV and all-cause death with a HR of 1.15 at full follow-up, Supplemental Table S2. There was a significantly better model prediction when adding AVPD to the other clinical risk factors assessed with the log-likelihood ratio (LR) test; χ^2^(1)=7.20, p=0.007 for CV-death, χ^2^(1)=12.03, p=0.001 for all-cause death.

GLS also remained as an independent predictor of CV and all-cause death at full follow-up (HR 1.10 for CV-death, HR 1.11 for all-cause death) Supplemental Table S2. Models with GLS were also better at predicting death compared to the same risk factors at full follow-up (LR test; χ2(1)=4.10, p=0.043 for CV death, χ2(1)=9.57, p=0.002 for all-cause death).

***Prognostic values of different AVPD measurement points***

Univariate Cox regression analyses for CV death for all six AV-plane locations are presented for the full follow-up in Figure S5. The highest HR of a single AVPD location was obtained from the septal locations in both the 3-and 4-chamber views (CV death: HR 1.16 and 1.17 and all-cause death: HR 1.18 and 1.16, respectively). Figure S5 shows the univariate analyses at all AVPD locations at full follow-up.

***Supplemental Tables***

***Table S1***. Values for the separate NYHA classes.

| **Variable** | **NYHA I** | **NYHA II** | **NYHA III** | **NYHA IV** |
| --- | --- | --- | --- | --- |
| AVPD [mm] | 9.5 ± 1.9 | 8.4 ± 2.3 | 7.4 ± 2.4 | 6.4 ± 1.9 |
| GLS [%] | -9.0 ± 3.1 | -8.5 ± 3.3 | -7.2 ± 2.9 | -6.3 ± 2.9 |
| EF [%] | 31.0 ± 5.2 | 28.1 ± 7.4 | 25.1 ± 7.6 | 22.9 ± 8.5 |

AVPD, atrioventricular plane displacement. GLS, global longitudinal strain. EF, ejection fraction. NYHA, New York Heart Association.

***Table S2.*** Univariate predictors at full-follow-up.

| ***Univariate predictors for HFrEF*** | | | | |
| --- | --- | --- | --- | --- |
| \|  \| \| --- \| | ***Cardiovascular death*** | | ***All-cause death*** | |
| ***Variable*** | ***HR (95% CI)*** | ***P value*** | ***HR (95% CI)*** | ***P value*** |
| Age, per 1 year | **1.08 (1.05-1.10)** | **<0.001** | **1.07 (1.05-1.09)** | **<0.001** |
| Sex, Male | 1.45 (0.82-2.57) | 0.20 | 1.37 (0.88.2,13) | 0.17 |
| BMI, per 1 kg/m^2^ | 0.96 (0.91-1.01) | 0.12 | **0.95 (0.91-0.99)** | **0.007** |
| Diabetes | **2.12 (1.26-3.54)** | **0.004** | **2.08 (1.39-3.12)** | **<0.001** |
| NYHA | **1.43 (1.05-1.94)** | **0.02** | 1.33 (1.04-1.70) | **0.02** |
| NT-proBNP, per 1000 ng/l | **1.13 (1.04-1.23)** | **0.006** | **1.15 (1.09-1.22)** | **<0.001** |
| eGFR, per 10 ml/min/1.73m^2^ | **0.77 (0.69-0.86)** | **<0.001** | **0.78 (0.71-0.86)** | **<0.001** |
| Smoking | 0.90 (0.48-1.68) | 0.75 | 0.97 (0.60-1.59) | 0.91 |
| Hypertension | 0.84 (0.49-1.68) | 0.51 | 1.29 (0.88-1.90) | 0.19 |
| Etiology, ICM | **3.08 (1.83-5.20)** | **<0.001** | **2.76 (1.86-4.11)** | **<0.001** |
| LGE presence | **3.45 (1.66-7.16)** | **0.001** | **2.67 (1.60-4.44)** | **<0.001** |
| Left atrial volume, per 10 ml | 1.05 (1.00-1.10) | 0.05 | 1.01 (0.98-1.05) | 0.49 |
| EF, per 1% | **1.03 (1.01-1.06)** | **0.03** | 1.02 (0.99-1.04) | 0.14 |
| EDVi, per 10 ml/m^2^ | 1.05 (1.00-1.09) | 0.052 | 1.01 (0.98-1.05) | 0.52 |
| Mean AVPD, per 1 mm | **1.15 (1.04-1.26)** | **0.004** | **1.12 (1.04-1.20)** | **0.003** |
| GLS, per 1% | **1.13 (1.05-1.22)** | **0.002** | **1.10 (1.03-1.17)** | **0.003** |
| TAPSE, per 1 mm | **1.05 (1.01-1.08)** | **0.011** | **1.06 (1.03-1.09)** | **<0.001** |

HR, hazard ratio. CI, confidence interval. BMI, body mass index. NYHA, New York Heart Association. BNP, brain natriuretic peptide. eGFR, estimated glomerular filtration rate. ICM, ischemic cardiomyopathy. LGE, late gadolinium enhancement. EF, ejection fraction. EDVi, end-diastolic volume indexed to body surface area. AVPD, atrioventricular plane displacement. GLS, global longitudinal strain. TAPSE, tricuspid annular plane systolic excursion.

***Table S3***. Multivariate modeling for HFrEF at full follow-up.

| **Multivariate Cox regression - Full follow-up** | ***Cardiovascular death*** | | ***All-cause death*** | |
| --- | --- | --- | --- | --- |
|  | ***HR (95% CI)*** | ***P value*** | ***HR (95% CI)*** | ***P value*** |
| **Model 1 - AVPD (n=287)** | ***LR test p=0.007*** | | ***LR test p=0.001*** | |
| Age, per 1 year | **1.07 (1.05-1.10)** | **<0.001** | **1.06 (1.04-1.08)** | **<0.001** |
| Sex, Male | 1.06 (0.58-1.93) | 0.86^2^ | 1.13 (0.71-1.79) | 0.62^2^ |
| BMI, per 1kg/m2 | 0.98 (0.93-1.04) | 0.54^3^ | **0.95 (0.91-0.99)** | **0.03** |
| Etiology, ICM | **2.19 (1.26-3.82)** | **0.005** | **1.80 (1.19-2.72)** | **0.006** |
| LGE presence | 1.75 (0.76-4.02) | 0.19^4^ | 1.35 (0.74-2.48) | 0.33^3^ |
| EF, per 1 % | 1.01 (0.96-1.05) | 0.90^1^ | 1.01 (0.97-1.04) | 0.97^1^ |
| EDVi, per 10 ml/m^2^ | **1.08 (1.02-1.14)** | **0.005** | 1.03 (0.98-1.07) | 0.26^4^ |
| Mean AVPD, per 1 mm | **1.15 (1.04-1.27)** | **0.001** | **1.15 (1.06-1.24)** | **0.001** |
| **Model 2 - GLS (n=283)** | ***LR test p=0.043*** | | ***LR-test p=0.002*** | |
| Age, per 1 year | **1.07 (1.04-1.10)** | **<0.001** | **1.06 (1.04-1.08)** | **<0.001** |
| Sex, Male | 1.09 (0.59-2.00) | 0.79^1^ | 1.17 (0.74-1.86) | 0.52^2^ |
| BMI, per 1kg/m2 | 0.98 (0.92-1.04) | 0.55^2^ | **0.95 (0.90-0.99)** | **0.03** |
| Etiology, ICM | **2.14 (1.22-3.75)** | **0.008** | **1.84 (1.21-2.79)** | **0.005** |
| LGE presence | 1.83 (0.79-4.27) | 0.16^4^ | 1.36 (0.73-2.51) | 0.34^3^ |
| EF, per 1 % | 1.02 (0.98-1.06) | 0.36^3^ | 1.02 (0.99-1.04) | 0.29^4^ |
| EDVi, per 10 ml/m2 | **1.07 (1.01-1.13)** | **0.02** | 1.00 (0.95-1.06) | 0.88^1^ |
| GLS, per 1 % | **1.10 (1.003-1.20)** | **0.043** | **1.11 (1.04-1.18)** | **0.002** |

BMI, body mass index. ICM, ischemic cardiomyopathy. LGE, late gadolinium enhancement. EF, ejection fraction. EDVi, end-diastolic volume indexed to body surface area. AVPD, atrioventricular plane displacement. GLS, global longitudinal strain. Log-likelihood ratio (LR)-tests assess the added prognostic values compared with nested models without AVPD (Model 1) and GLS (Model 2). Numbers in superscript represents the last step before elimination for that variable.

| **Multivariate Cox regression** | ***Cardiovascular death*** | | ***All-cause death*** | |
| --- | --- | --- | --- | --- |
|  | ***HR (95% CI)*** | ***P value*** | ***HR (95% CI)*** | ***P value*** |
| **TAPSE – 5-year** | ***LR test p=0.046*** | | ***LR test p=<0.001*** | |
| Age, per 1 year | **1.06 (1.03-1.10)** | **<0.001** | **1.04 (1.02-1.07)** | **0.002** |
| Sex, Male | 0.77 (0.30-1.96) | 0.58^2^ | 0.76 (0.52-2.49) | 0.76^1^ |
| BMI, per 1kg/m2 | 0.95 (0.87-1.03) | 0.20^5^ | **0.94 (0.88-1.01)** | **0.07** |
| Etiology, ICM | 1.56 (0.69-3.49) | 0.28^4^ | 1.44 (0.76-2.72) | 0.26^4^ |
| LGE presence | 1.37 (0.41-4.56) | 0.61^1^ | 1.31 (0.51-3.34) | 0.57^2^ |
| EF, per 1 % | **1.07 (1.02-1.11)** | **0.003** | 1.03 (0.99-1.06) | 0.11^5^ |
| EDVi, per 10 ml/m^2^ | 1.05 (0.96-1.15) | 0.26^3^ | 1.03 (0.96-1.11) | 0.42^3^ |
| Mean TAPSE, per 1 mm | **1.07 (1.01-1.12)** | **0.046** | **1.08 (1.04-1.13)** | **<0.001** |

***Table S4***. Multivariate modeling with TAPSE at 5-year follow-up.

BMI, body mass index. ICM, ischemic cardiomyopathy. LGE, late gadolinium enhancement. EF, ejection fraction. EDVi, end-diastolic volume indexed to body surface area. TAPSE, tricuspid annular plane systolic excursion. Log-likelihood ratio (LR)-tests assess the added prognostic values compared with nested models without TAPSE. Numbers in superscript represents the last step before elimination for that variable.

***Supplemental Figure
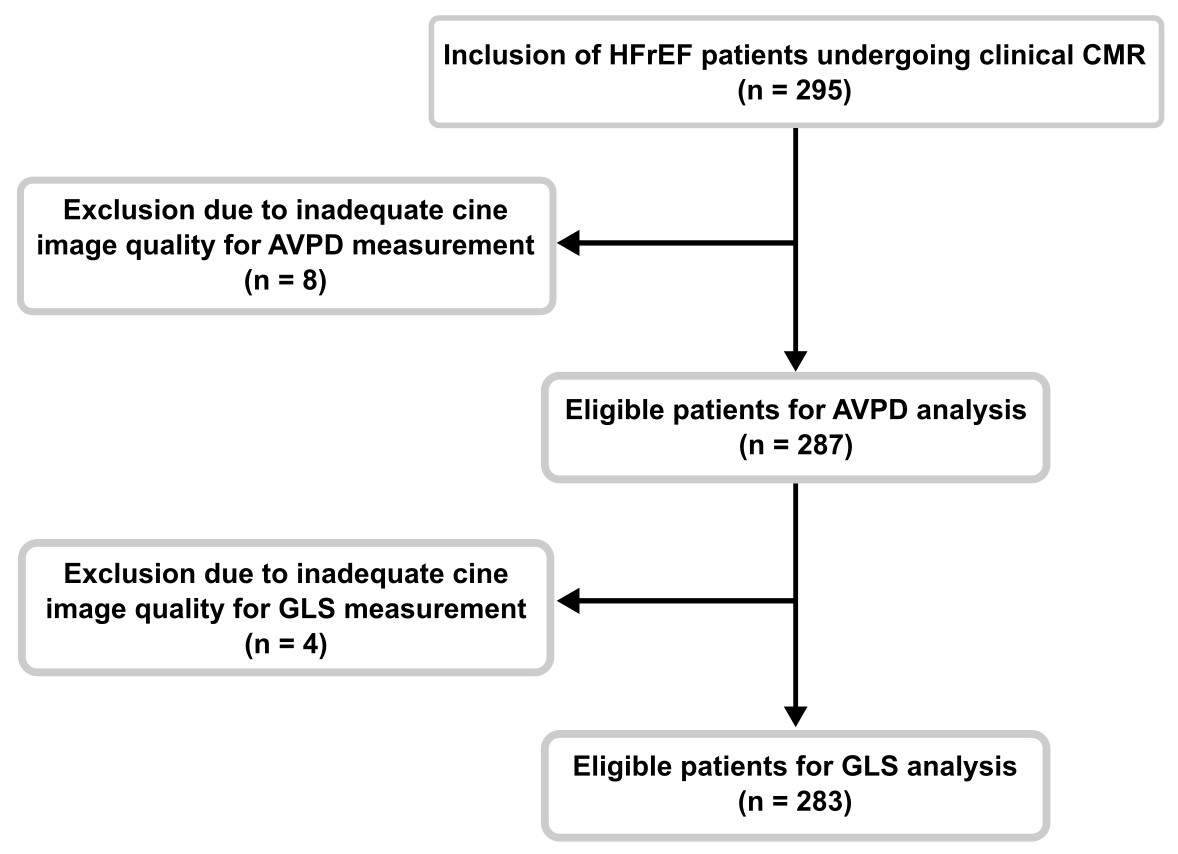
***

**Figure S1.** *Flowchart of the study cohort*. HFrEF, Heart failure with reduced ejection fraction. AVPD, atrioventricular plane displacement. GLS, global longitudinal strain.


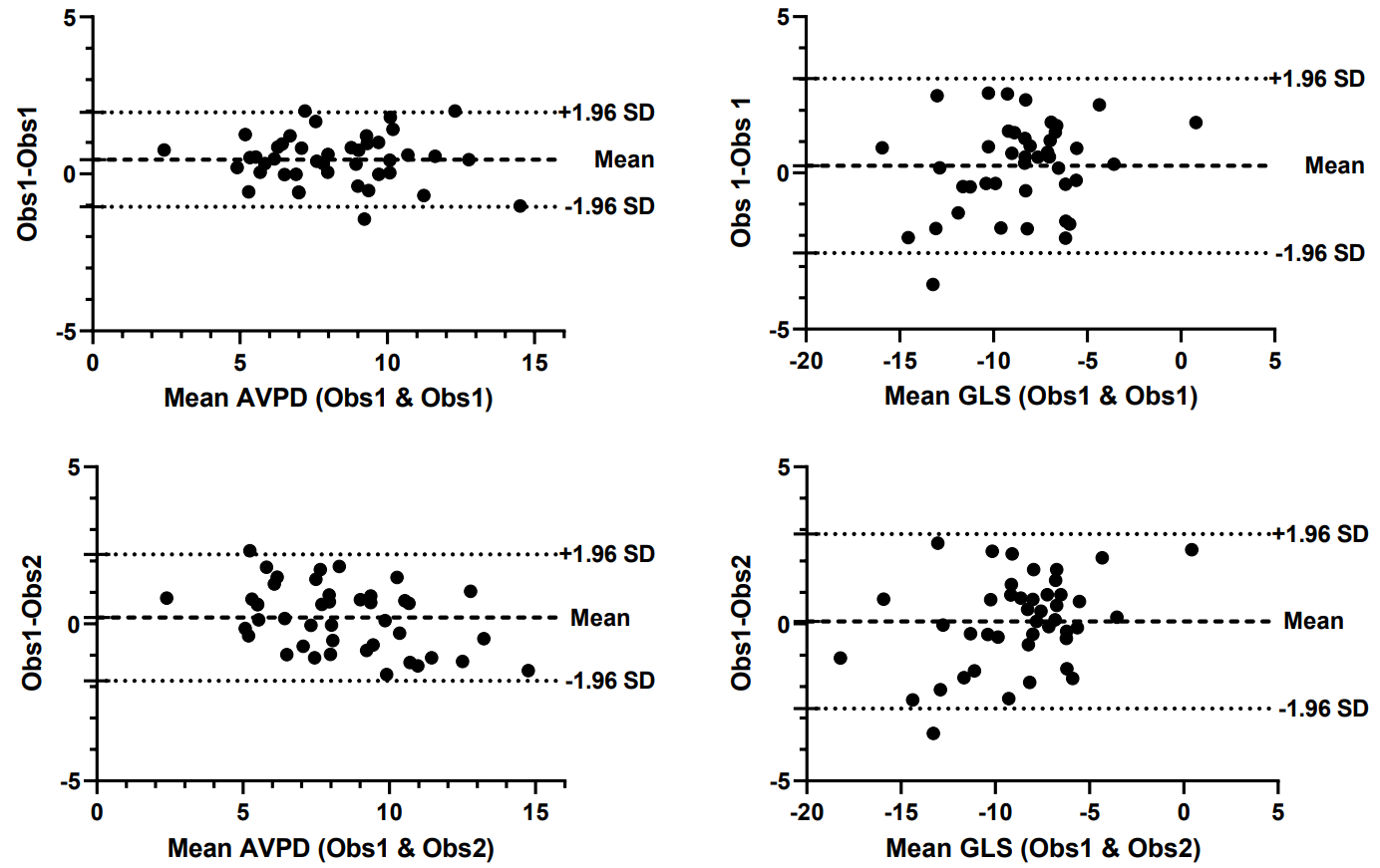


**Figure S2.** Bland & Altman plots of the variability of AVPD and GLS measurement between the same and two different observers.


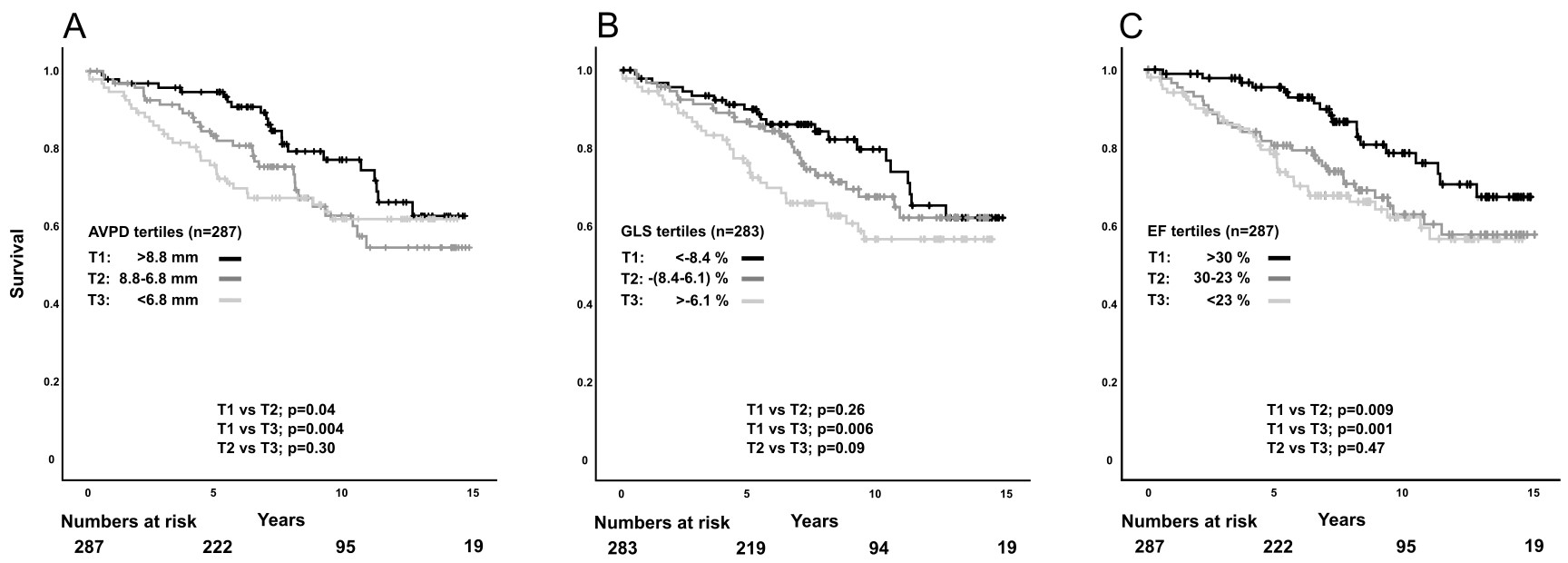


**Figure S3.** Kaplan-Meier curves showing survival from cardiovascular death stratified by tertiles for (A) atrioventricular plane displacement (AVPD), (B) global longitudinal strain (GLS) and (C) ejection fraction (EF) for the full follow-up. T1, upper tertile. T2, middle tertile. T3, lower tertile.


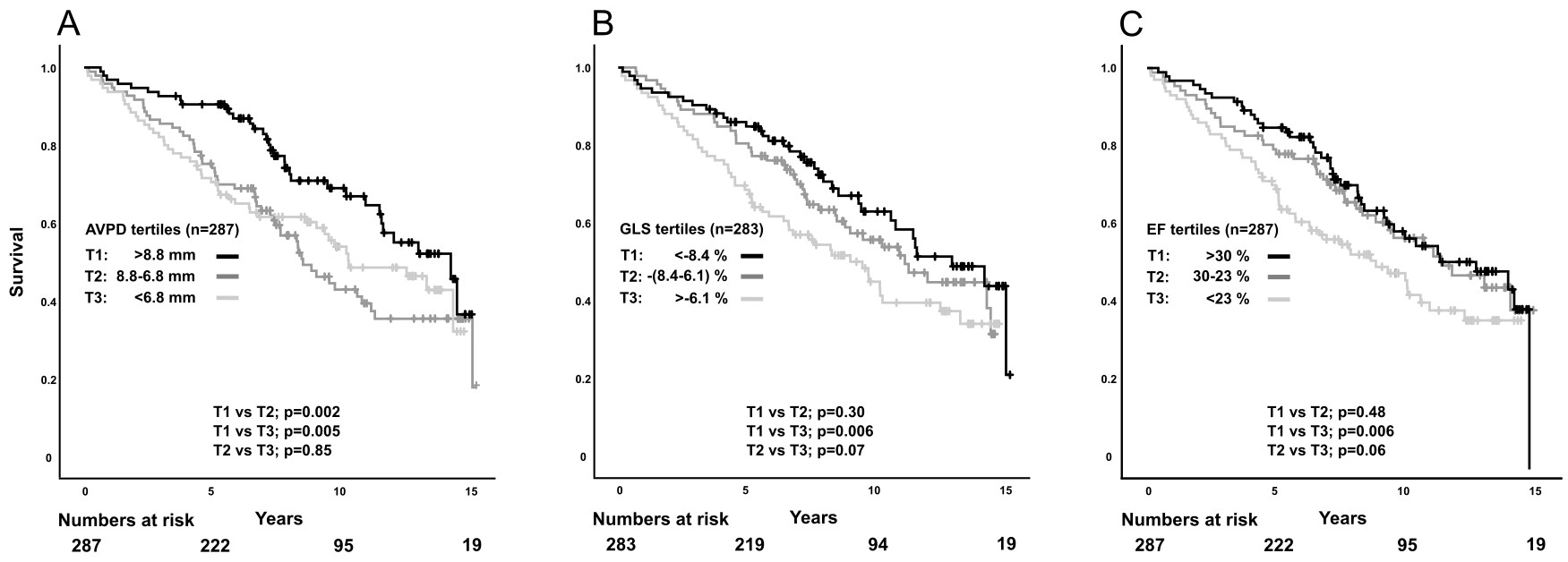


**Figure S4.** Kaplan-Meier curves showing survival from all-cause death stratified by tertiles for (A) atrioventricular plane displacement (AVPD), (B) global longitudinal strain (GLS) and (C) ejection fraction (EF) for the full follow-up. T1, upper tertile. T2, middle tertile. T3, lower tertile.


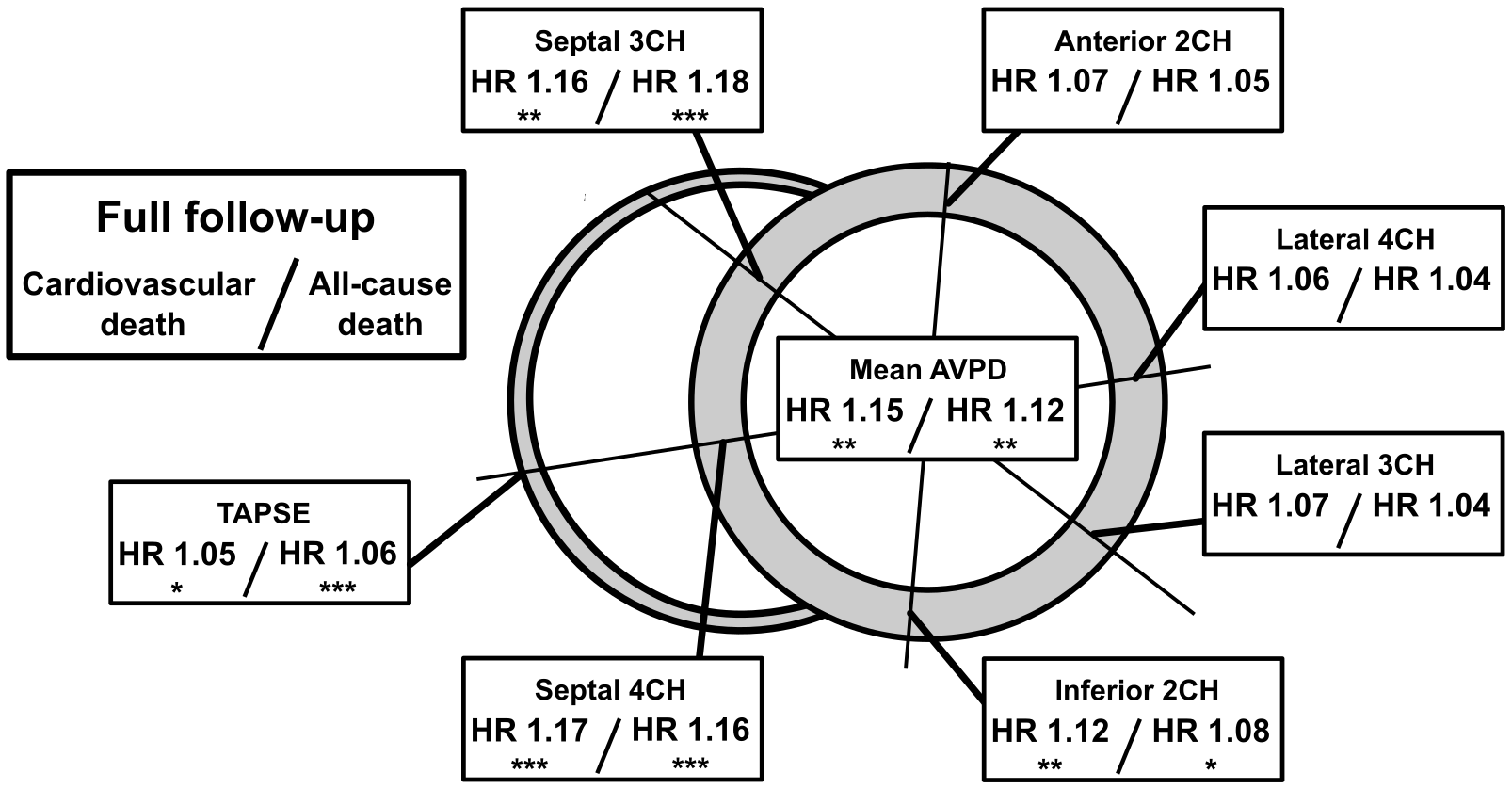


**Figure S5**. Univariate Cox regression analyses of the six atrioventricular plane displacement (AVPD) locations. The short-axis slice illustrates hazard ratios (HR) for cardiovascular death and all-cause death at full follow-up for each location. Septal and average locations indicate higher HR values than lateral, anterior and inferior points. Asterisks represent *p<0.05, **p<0.01, ***p<0.001. TAPSE, tricuspid annular plane systolic excursion.
